# Generation of human antibody fragments against *Streptococcus mutans *using a phage display chain shuffling approach

**DOI:** 10.1186/1472-6750-5-4

**Published:** 2005-01-25

**Authors:** Michael B Kupper, Michael Huhn, Holger Spiegel, Julian KC Ma, Stefan Barth, Rainer Fischer, Ricarda Finnern

**Affiliations:** 1 Fraunhofer Institute for Molecular Biology and Applied Ecology, 52074 Aachen, Germany; 2 Technical University Aachen, Department of Molecular Biotechnology, 52074 Aachen, Germany; 3 Department of Cellular and Molecular Medicine, Molecular Immunology Unit, St. George's Hospital Medical School, London SW17 ORE, UK

## Abstract

**Background:**

Common oral diseases and dental caries can be prevented effectively by passive immunization. In humans, passive immunotherapy may require the use of humanized or human antibodies to prevent adverse immune responses against murine epitopes. Therefore we generated human single chain and diabody antibody derivatives based on the binding characteristics of the murine monoclonal antibody Guy's 13. The murine form of this antibody has been used successfully to prevent *Streptococcus mutans *colonization and the development of dental caries in non-human primates, and to prevent bacterial colonization in human clinical trials.

**Results:**

The antibody derivatives were generated using a chain-shuffling approach based on human antibody variable gene phage-display libraries. Like the parent antibody, these derivatives bound specifically to SAI/II, the surface adhesin of the oral pathogen *S. mutans*.

**Conclusions:**

Humanization of murine antibodies can be easily achieved using phage display libraries. The human antibody fragments bind the antigen as well as the causative agent of dental caries. In addition the human diabody derivative is capable of aggregating *S. mutans in vitro*, making it a useful candidate passive immunotherapeutic agent for oral diseases.

## Background

Dental caries is one of the most common infectious diseases of humans. The main causative agent is a group of streptococcal species collectively described as the mutans streptococci [1]. *Streptococcus mutans *has been identified as the major etiological agent of the disease. Unlike many other diseases, dental caries is as prevalent in the West as it is in developing countries, and therefore attracts significant interest from medical and dental authorities as well as pharmaceutical companies. The first step in the initiation of infection is the attachment of the bacterium to a specific receptor, and this is an ideal point for intervention. Two groups of proteins from mutans streptococci represent primary candidates for a human caries vaccine: i) glucosyltransferase enzymes, which synthesize adhesive glycans and allow microbial accumulation, and ii) cell surface fibrillar proteins that mediate adherence to the salivary pellicle [2]. The bacterial adhesin SAI/II [3], a surface-displayed protein with a molecular mass of 190 kDa, plays an important role in the initial attachment of *S. mutans *to the tooth surface. Antibodies recognizing this protein prevent colonization of the buccal cavity by the bacterium and could be developed as a vaccine against dental caries. The most suitable vaccination strategy would be passive immunization, in which monoclonal antibodies or fragments thereof are applied to the tooth surface e.g. using toothpaste, mouthwash or chewing gum. This would make active immunization with the *S. mutans *adhesin unnecessary.

The murine monoclonal antibody Guy's 13 [4] which specifically recognizes the SAI/II protein of *S. mutans *and *Streptococcus sobrinus *has been used successfully to prevent *S. mutans *colonization and the development of dental caries in non-human primates [5]. The antibody also prevented bacterial colonization in human clinical trials [6,7]. However, like other murine antibodies, a major limitation in clinical applications may be the human anti-mouse antibody response (HAMA), which can increase the rate of clearance and initiate allergic reactions [8]. The problems associated with murine antibodies can be overcome by replacing murine sequences with their human counterparts, e.g. by chimerization [9], CDR grafting [10] and guided selection using phage display technology [11]. Furthermore, the use of antibody fragments rather than whole antibodies also removes some of the constant regions that may provoke an immune response.

There has been a growing interest in the use of single-chain fragment variable (scFv) antibodies, in which the variable regions of the heavy and light chains are combined in the same polypeptide chain (Huston, 1988 #2785). The advantages of such derivatives are that they can be expressed as single transgenes in various hosts, they fold spontaneously to adopt the correct tertiary structure, and their small size facilitates tissue penetration. The scFv has the heavy and light chain variable regions joined by a flexible peptide linker allowing the two domains to interact, forming a univalent antibody. Alternatively, diabodies have the same structure but the two domains are joined by a shorter, less-flexible linker, forcing dimerization and the formation of divalent antibodies (Holliger, 1993 #3498).

We have generated human derivatives of the murine Guy's 13 antibody using a chain-shuffling approach based on human antibody variable gene phage-display libraries. We have taken the variable gene regions of the original Guy's 13 monoclonal antibody and created human scFv and diabody derivatives by chain shuffling in human phage-display libraries. Firstly, the heavy chain variable gene of the Guy's 13 construct was introduced into a naïve human light chain phage display library to select human light chains that, in combination with the murine heavy chain, showed binding specificity for the SAI/II antigen. Once such chimeric antibodies had been selected, the murine heavy chain gene was replaced with the human counterpart, by introducing the selected human light chain genes into a human heavy chain phage display library.

The resulting clones were expressed in bacteria and tested for specificity in ELISAs using both SAI/II antigen and whole *S. mutans*. The stepwise procedure for generating human antibody chains allows the advantages of scFv and diabody antibody fragments to be exploited without suffering the negative effects of non-human antibodies in a clinical setting. The human antibody fragments were expressed in bacteria as scFv and diabody derivatives and used to aggregate *S. mutans in vitro*. The diabodies were able to aggregate the bacteria and therefore have the potential to be developed as therapeutic agents to treat and/or prevent dental caries.

## Results

### Human recombinant scFv antibodies against SAI/II

**Figure 1:**
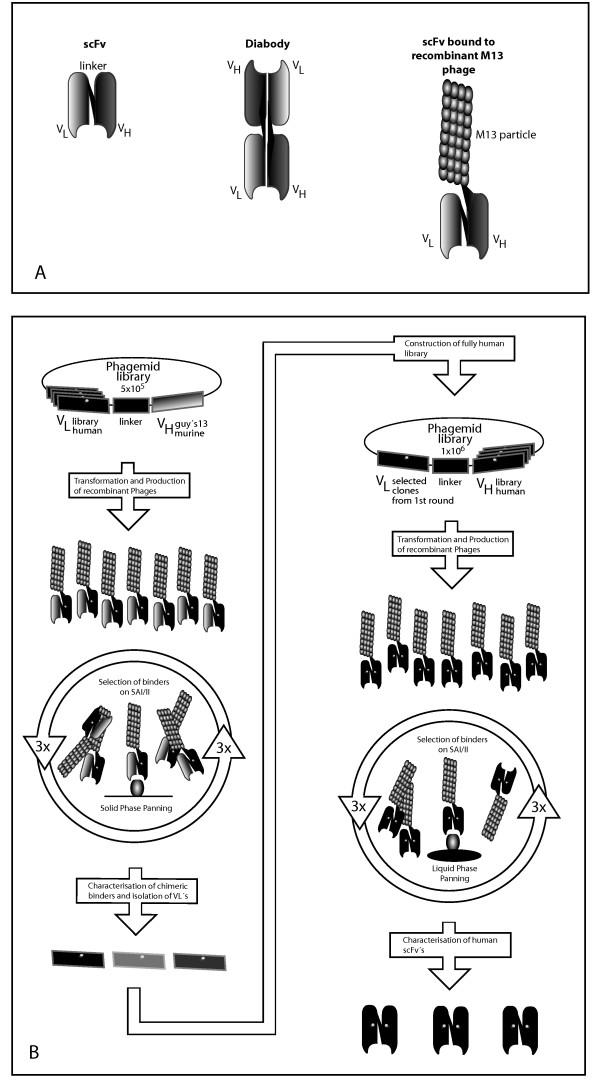
Construction and selection of human SAI/II specific antibody fragments. A: Antibody formats: scFv, in which the variable regions of the antibody heavy and light chains are combined in the same polypeptide chain joined by a (Gly_4_Ser)_3 _amino acid linker. The diabody construct consists of the antibody variable heavy and light chain domains linked by a ten-amino-acid linker (TGGGGSSSAL), forcing the expressed domains to attach to a complementary chain in solution to create two antigen-binding sites. scFv presentation as a protein3 fusion of the M13 phage. B: Chain shuffling approach: The variable antibody domain is cloned into a phagemid vector containing a human antibody variable domain library. The selection on SAI/II antigen was carried out for three consecutive rounds. Light chains participating in binding to SAI/II were identified and cloned into a phagemid vector containing a human antibody heavy domain library. Selection on SAI/II antigen for three rounds yielded fully human SAI/II specific scFv antibody fragments.

**Figure 2:**
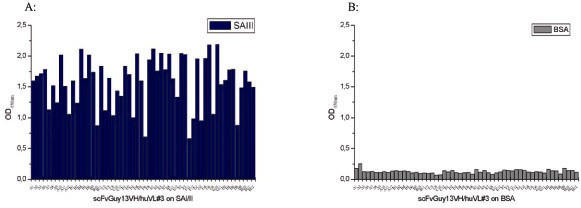
Specificity of chimeric scFv (mGuy13VH/huVL) ELISA reactivity of overnight induced bacterial culture supernatant containing chimeric scFv (mGuy13VH/huVL) after 3 rounds of selection on SAI/II antigen. A: 96 individual scFvs on SAI/II coated ELISA plates. B: the same 96 scFvs on BSA coated ELISA plates

**Figure 3:**
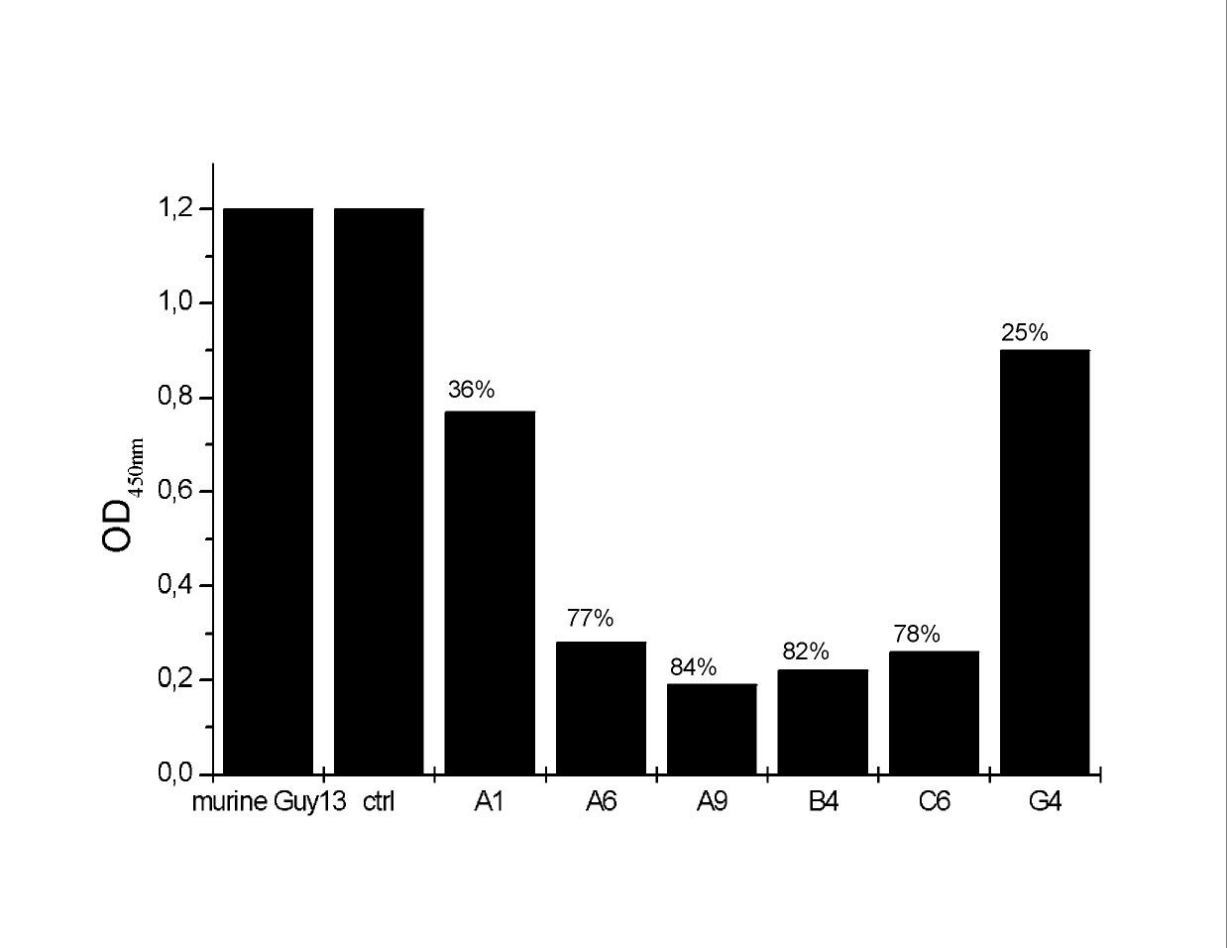
Inhibition of chimeric scFv (mGuy13VH/huVL) to SAI/II by the monoclonal Ab Guy13. Chimeric scFv were allowed to bind to SAI/II followed by replacement with an access of mAb Guy 13. The experiments were carried out in duplicate and the mean values are shown. Bound scFv were detected via their MYC-tag. The murine mAb Guy13 bound to the SAI/II. The scFv Guy13 was not replaced by an unrelated mAb (ctrl.) Binding of A1 and G4 was only inhibited by 36% and 25%, respectively indicating that these scFv might recognize a different epitope than mAb Guy13. The binding of chimeric scFv A6, A9, B4, and C6 were inhibited between 77–84% indicating that they recognize the same epitope as mAb Guy13.

Human scFv antibody fragments based on the murine monoclonal antibody Guy's 13 were constructed using two consecutive rounds of variable-domain shuffling and phage-library selection (Figure 1). First, a chimeric scFv was generated by amplifying the murine Guy's 13 heavy chain variable region, and inserting it into a human light chain variable region phage display library. The resulting phage display library had a complexity of 5 × 10^5^. Single chain Fv antibody fragments with appropriate binding activities were selected on purified, immobilized SAI/II antigen. Three rounds of selection were carried out and unique candidate antibodies were identified by ELISA (Figure 2). Subsequent sequencing yielded five antibody fragments (chimscFvA1, chimscFvA6, chimscFvA9, chimscFvB4, and chimscFvG4). Sequencing of the human variable genes showed that two of the clones chimscFvA6, chimscFvB4 belonged to family Vκ1, clone chimscFvA6 was homologous to HK137 and chimscFvB4 was homologous to the L12 germline gene family. ChimscFvA9 belonged to family Vκ4 DPκ24. ChimscFvA1 and chimscFvG4 belonged to family Vλ3 DPL16) (data not shown). Inhibition ELISA showed that the binding of all six chimeric scFvs to SAI/II could be inhibited by the murine monoclonal antibody Guy's 13. The binding of chimeric scFvs A6, A9, B4 and C6 was inhibited by approximately 80%, suggesting that epitope recognition was maintained (Figure 3). The binding of the chimeric scFvs A1 and G4 was only inhibited by approximately 30%, suggesting that these antibodies recognized a different epitope.

**Figure 4:**
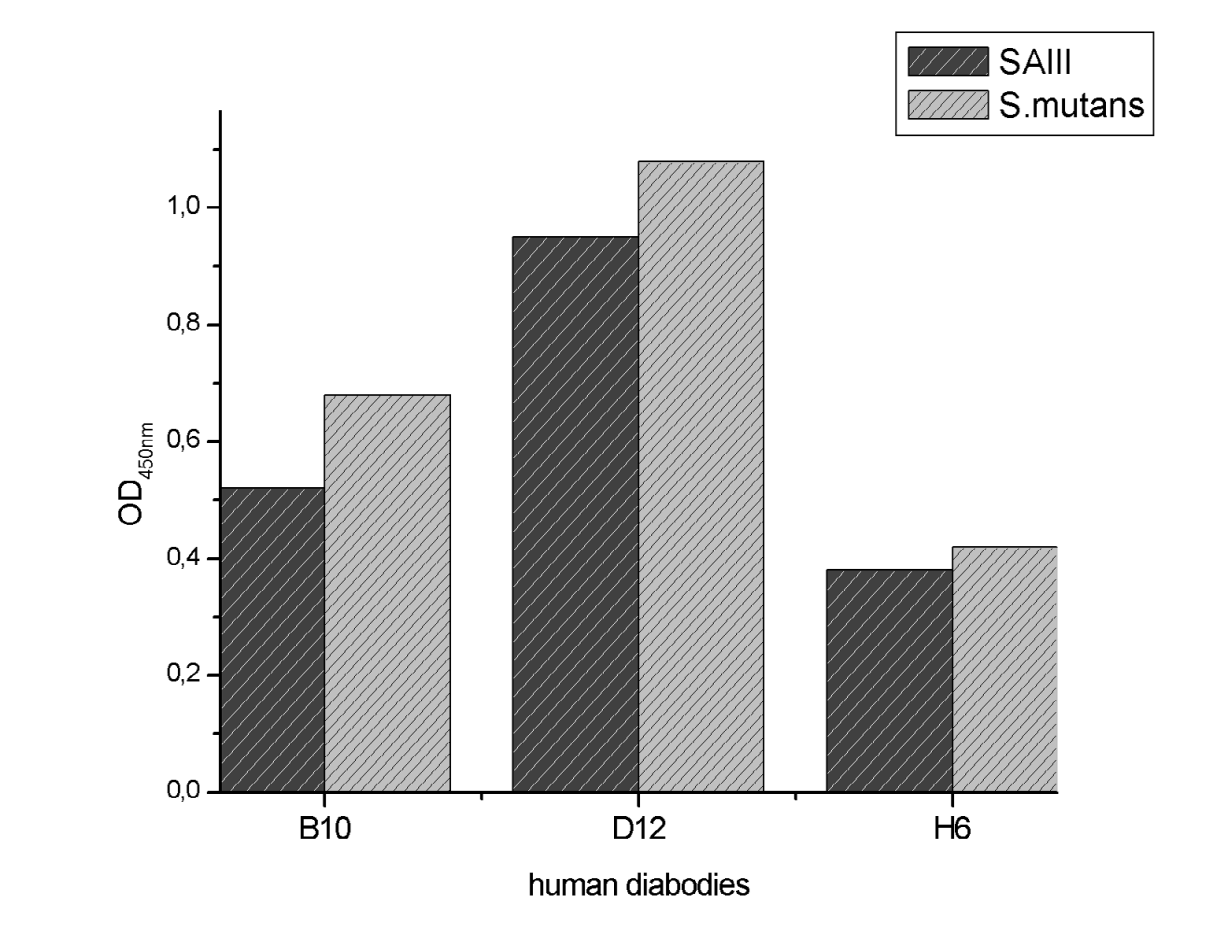
Binding of human diabodies to SAI/II and *S. mutans *coated ELISA plates. One μg SAI/II or 10 ml *S. mutans *culture were immobilized on the ELISA plate. Binding of 1 μg human diabody B10, D12 and H6 was detected via the His_6_tag. The experiments were carried out in duplicate and the mean values are shown. The three human diabodies recognize the SAI/II antigen in the purified form as well as in the context of the bacterial surface.

The selected human V_L _genes were introduced into a human V_H _library (complexity 8 × 10^8^) and a combinatorial library with a complexity of 1 × 10^6 ^was established. Three rounds of selection were carried out in solution using SAI/II antigen coupled to paramagnetic beads. Eleven human scFvs were identified by ELISA (data not shown). Subsequent sequence analysis identified three human scFvs: clones huscFv B10, huscFv D12 and huscFv H6. Table 1 shows the amino acid sequences of the human scFv antibody fragments. The human V_L _domain in chimeric scFv A6 (Vκ1 HK137) was selected in combination with two different human variable heavy chains, giving human scFvs B10 and H6, respectively. The V_H _domain of human scFv B10 is homologous to V_H_1 family DP10, and the V_H _domain of human scFv H6 is homologous to V_H_3 family DP35. The human V_L _domain in chimeric scFv B4 (Vκ1 L12) was selected in combination with one human variable heavy chain giving the human scFv D12. The V_H _domain of human scFv D12 is homologous to VH5 family DP73. Figure 4 shows the binding of the three human scFvs to the SAI/II antigen and the pathogenic bacterium *S. mutans*. Inhibition ELISA showed that the binding of all three human scFvs to SAI/II was inhibited by Guy's 13, suggesting that epitope recognition was maintained.

### Generation of human diabodies and agglutination of *S. mutans*

**Figure 5:**
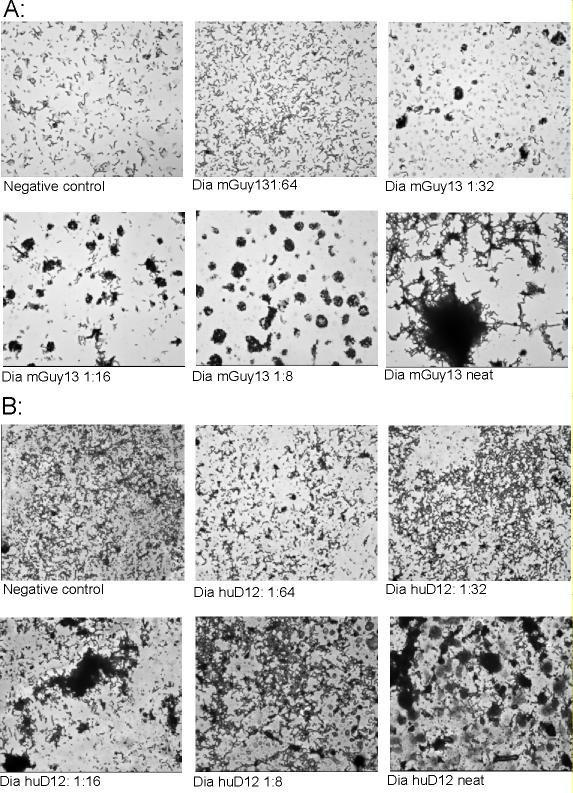
Bacterial agglutination assay. *S. mutans *was incubated with serial dilutions of diabody for 1 h at 37°C followed by counterstaining with Gram solution and analysis by microscopy. A: Murine diabody mGuy13. B: Human diabody D12. Neg. ctrl.: Unrelated human diabody

Recombinant antibody fragments can be engineered to assemble into stable multimeric oligomers of high binding avidity and specificity [12]. A scFv molecule joined by a linker of 3–12 residues cannot fold into a functional Fv domain and instead associates with a second scFv molecule to form a bivalent dimer (diabody, approx. 60 kDa). For the cross-linking of cell surface antigens, at least two binding domains are necessary. The diabody is the smallest bivalent antibody molecule that can fulfill this prerequisite. Through reduced off-rates, which result from multiple binding to two target antigens and to rebinding when one Fv dissociates, the diabody is suitable to facilitate specific agglutination of bacteria. We constructed human diabodies by isolating the variable heavy and light chain genes from human scFvs B10, D12 and H6 and murine scFv Guy's 13, amplified by PCR (Table 2) and inserting them in two consecutive steps into the vector pHenIXdia, containing a 10 amino acid residue linker. The integrity of the clones was confirmed by sequencing and the binding activity was demonstrated by ELISA using both the SAI/II antigen and *S. mutans *cells (Figure 4). Ma et al. [7] reported that bivalent binding of the murine Guy's 13 is required for protection against dental caries, since the F(ab')2 derivative was protective but not the monovalent Fab fragment. *S. mutans *was aggregated in a dose dependent manner when grown in the presence of murine diabody Guy's 13 and human diabody D12 (Figure 5A and 5B).

## Discussion

Antibodies recognizing and neutralizing the oral pathogen *S. mutans *provide a novel approach for the control and prevention of dental caries. A monoclonal antibody that binds specifically to the SAI/II surface adhesin of *S. mutans *was isolated by Smith et al. (20) and has been expressed in plants as a secretory IgA (sIgA) [13]. In ongoing Phase II clinical trials, this recombinant antibody has been shown to prevent recolonization of the mouth by *S. mutans *when coated onto the teeth and gums following eradication of the bacteria. The sIgA is probably the most appropriate format for the topical application of antibodies that inhibit the colonization of the tooth surface by *S. mutans *because this is the predominant form of antibody naturally found in the saliva. However, each sIgA comprises ten polypeptide chains of four different types making it difficult to produce on a large scale in conventional production systems. The more convenient diabody antibody derivatives, which can be expressed in large quantities in microbial culture systems, may be more suitable for the type of production scales that would be required for the routine control of dental caries using this strategy.

As a first step in this direction, we have taken the variable gene regions of the original Guy's 13 monoclonal antibody and created human scFv and diabody derivatives by chain shuffling in human phage-display libraries. The heavy chain variable gene of the Guy's 13 construct was introduced into a naïve human light chain phage display library to select human light chains that, in combination with the murine heavy chain, showed binding specificity for the SAI/II antigen. Once such chimeric antibodies had been selected, the murine heavy chain gene was replaced with the human counterpart, by introducing the selected human light chain genes into a human heavy chain phage display library.

The resulting clones were expressed in bacteria and tested for specificity in ELISAs using both SAI/II antigen and whole *S. mutans*. The stepwise procedure for generating human antibody chains allows the advantages of scFv and diabody antibody fragments to be exploited without suffering the negative effects of non-human antibodies in a clinical setting. Small scFv and diabody antibody fragments are easy to express in large quantities, they penetrate tissues easily and they lack the constant domains that promote often-unwanted and usually superfluous effector functions. However, where such antibodies are murine in origin, they can provoke an immune reaction in the human host, leading to rapid clearance and poor efficacy during long-term treatment. Since dental caries tends to be chronic rather than acute, murine antibodies would be of little benefit to patients in the long term.

Two drawbacks of scFvs compared to the ideal sIgA format are monovalency and instability. ScFvs are monovalent because the heavy and light chains are joined by a flexible peptide linker, which allows the two domains to fold and interact with each other. We have addressed this problem by converting the scFv antibodies into diabodies, which is achieved by shortening the linking peptide and forcing the heavy and light chain variable domains to seek interaction partners as part of a dimer. As a consequence of this interaction, the diabody is bivalent like the parent immunoglobulin, and therefore has increased binding avidity. We showed that the bivalent binding of the diabody antibody constructs leads to agglutination of *S. mutans*.

The problem of decreased stability may be more difficult to address, because the efficacy of scFv and diabody molecules used to treat dental caries will depend largely on their persistence and effective concentration. Secretory IgAs include a secretory component, which protects the antibody from proteolytic degradation in the saliva. One possible solution is to include this secretory component in any scFv or diabody format through the use of further gene fusion strategies. However, it is envisaged that antibodies for the prevention of dental caries will be administered in the form of toothpaste or mouthwash, or perhaps chewing gum, which will allow the treatment to be refreshed at regular intervals.

## Conclusions

We have shown that the humanization of a murine monoclonal antibody can be easily achieved using a chain-shuffling approach based on scFv antibody phage display libraries. The human antibody derivatives of the murine Guy's 13 antibody can be expressed and isolated from bacteria, recognize SAI/II and *S. mutans *with great specificity, and can successfully aggregate *S. mutans *cells in the dimeric form in a dose dependent manor. These recombinant therapeutic proteins therefore represent the first step towards an inexpensive and convenient general treatment for dental caries.

## Methods

### Propagation of *S. mutans*

*S. mutans *20523 serological group c was purchased from DSMZ (Braunschweig, Germany) and grown in an S2 containment laboratory in trypticase soy yeast extract medium (30 g l^-1 ^trypticase soy broth, 3 g l^-1 ^yeast extract, pH 7.0–7.2) at 37°C for 2 d prior to use.

### Cloning the *S. mutans spa*P gene encoding surface antigen SAI/II

Nucleotides 214–3048 of the *spa*P gene [14], which encodes the SAI/II antigen, were removed from pUC18 as a *Sfi*I/*Not*I fragment and inserted into the bacterial expression vectors pCantab5E (Pharmacia) and pSin1 [15] which had been digested with the same enzymes followed by transformation into *E. coli *TG1. The pCantab5E vector contained an additional sequence encoding the E-tag, facilitating the detection of expressed proteins using the monoclonal antibody 5E (Pharmacia). The pSin1 vector similarly contained sequences encoding a MYC-tag, facilitating detection with the murine monoclonal antibody 9E10 (ATCC CRL 1729), and a His_6 _tag, allowing purification of expressed proteins by immobilized metal-chelate affinity chromatography (IMAC) and detection using a murine Penta-HIS antibody (Qiagen). SAI/II expressed using pSin1 was used for the selection of antibodies from phage-display libraries. SAI/II expressed in pCantab5E was used for enzyme-linked immunosorbent assays (ELISAs).

### Coating paramagnetic beads with SAI/II

For the selection of phage-display antibodies, 250 μl of phosphate-buffered saline (PBS)-washed Dynabeads (Dynal Biotech GmbH) was resuspended in 500 μl 0.1 M phosphate buffer (pH 7.4) and mixed gently for 2 min. The beads were collected with a magnet, the supernatant discarded and the beads resuspended in 250 μl of the same buffer, followed by the addition of 500 μl SAI/II antigen (1 mg/ml). After incubation for 16 h at 37°C with slow tilt rotation, the beads were collected with a magnet and the supernatant was discarded. The coated beads were washed four times, twice with 0.13 M NaCl, 1% milk powder in 0.01 M phosphate buffer (pH 7.4) for 5 min at 4°C, once with 0.2 M Tris-HCl (pH 8.5) for 4 h at 37°C and again in the same buffer for 5 min at 4°C.

### Cloning scFv Guy's 13 in pSin1

The variable region genes of the heavy and light chain of the murine monoclonal antibody Guy's 13 were amplified using oligonucleotide primers LMB3 (5' CAG GAA ACA GCT ATG AC 3') and fdSeq 1 (5' GAA TTT TCT GTA TG/AG GG 3') followed by digestion with *Sfi*I and *Not*I. The products were inserted into the phagemid vector pSin1, which had been treated with the same enzymes, and the recombinant vector was introduced into *E. coli *strain TG1.

### Construction and selection of human SAI/II-specific scFv antibodies

Figure 1 shows the schematic scheme of the construction and selection of human SAI/II specific scFv antibody approach. The variable heavy chain antibody domain of the murine antibody Guy's 13 was cloned as an *Sfi*I/*Sal*I fragment in the bacterial expression vector pHenIX containing a light-chain antibody phage-display library derived from naïve human peripheral blood lymphocytes (8 × 10^8^; R. Finnern, unpublished data). This vector is based on the phagemid vector pHen1 [16] designed to express antibody fragments as N-terminal fusions with the minor coat protein of filamentous bacteriophage M13. An amber stop codon between the two fusion partners allows the expression of both soluble antibody fragments and phage particles displaying recombinant antibodies. The recombinant vectors were introduced into *E. coli *strain TG1.

Three rounds of selection were carried out using immobilized SAI/II antigen as described by Marks et al. [17]. Elution was achieved using the monoclonal antibody Guy's 13 to select binders recognizing the same epitope. The expression of soluble scFvs was performed as described by Marks et al. [17] and scFvs specific for the SAI/II antigen were identified by ELISA using SAI/II antigen.

The selected variable antibody domain genes of the shuffled human light chains were cloned as *Apa*LI and *Not*I fragments in pHenIX containing a human variable heavy chain library (8 × 10^8^; R. Finnern, unpublished data) and introduced into *E. coli *TG1. This was achieved by PCR amplification of the human light chain genes using primers Vκ4 *Apa*LI (5'-TGAGCACACAGTGCACTCGACATCGTGATGACCCAGTCTCC-3'), Vκ1 *Apa*LI (5'-TGAGCACACAGTGCACTCGACATCCAGATGACCCAGTCTCC-3') and Jκ1 *Not*I (5'-GAGTCATTCTCGACTTGCGGCCGCACGTTTGATC/TTCCAC/GCTTGGTCCC-3').

Three rounds of selection were carried out using SAI/II antigen immobilized on Dynabeads. Briefly, 150 μg of SAI/II-coated beads was blocked for 1 h with 2 ml 2% milk powder. The beads were collected with a magnet, washed in PBS and incubated with the antibody phage display library for 1 h on a turntable. The beads were washed 15 times with PBS containing 0.05% Tween 20 and 15 times with PBS to remove unbound phage. Bound phage were eluted with 100 μl 100 mM triethanolamine for 10 min on a turntable followed by neutralization in 200 μl 1 M Tris-HCl (pH 8.0). Eluted phage were used to infect exponentially growing *E. coli *TG1 and grown overnight at 30°C on TYE plates containing 100 μg ml^-1 ^ampicillin, 1% glucose. Selection, phage rescue and induction of soluble scFv expression were carried out as described by Marks et al. [17]. Antigen-specific human scFvs were identified by ELISA using the SAI/II antigen.

### Propagation of phage display antibody libraries

One litre of 2× TY (supplemented with 100 μg ml^-1 ^ampicillin, 1% glucose) was inoculated with an aliquot of the phage antibody library glycerol stock. The rescue and induction of the phage was carried out essentially as described in Marks et al. [17]. Phagemid rescue was carried out by the addition of 10^10 ^units of helper phage VCSM13 (Pharmacia) to the growing phage antibody library. The culture medium was changed to 2× TY containing 100 μg ml^-1 ^ampicillin and 25 μg ml^-1 ^kanamycin and incubated on an orbital shaker overnight at 30°C and 250 rpm. Phage were purified twice by polyethylene glycol (PEG) precipitation (20% PEG, 2.5 M NaCl) and resuspended in a final volume of 2 ml PBS. The phage were stored at 4°C until further use.

### DNA sequencing

The number of unique clones was determined by PCR amplification of the recombinant antibody inserts using primers LMB3 (5' CAG GAA ACA GCT ATG AC 3') and fdSeq 1 (5' GAA TTT TCT GTA TG/AG GG 3') followed by digestion with the restriction enzyme *Bst*NI (New England Biolabs). The variable antibody genes from two clones of each restriction pattern were analyzed by PCR cycle sequencing using infrared-labeled primers according to the manufacturer's instructions (Licor). Sequencing reactions were carried out on a Licor automated DNA sequencer (4000 L) and the sequences were analyzed using Sequencher 3.1 (Gene Codes Corporation). The sequences of the V_H _and V_L _genes were compared with the germline sequences in the V-BASE database (Tomlinson et al., MRC Centre for Protein Engineering, Cambridge, UK).

### Construction of diabodies

The construction of diabodies [18] was carried out by PCR amplification of the variable heavy and light chain antibody regions of the human scFv clones and subcloning these in vector pHenIXdia. The diabody constructs consisted of the variable heavy and light chain antibody domains linked by a ten-amino-acid linker (TGGGGSSSAL), forcing the expressed domains to attach to a complementary chain in solution to create two antigen-binding sites. The primers used for the construction of the diabody antibody format are listed in Table 1.

### Upscaled production of recombinant proteins

Recombinant proteins were recovered from the bacterial periplasm following induction with 0.5 mM final concentration of isopropyl-β-D-galactopyranoside (IPTG) for 3–4 h at 30°C [19]. After centrifugation (4000 × g, 4°C, 30 min), the pellet was resuspended in 10 ml 30 mM Tris-HCl (pH 8.2) containing 20% sucrose, 1 mM ethylenediaminetretaacetic acid (EDTA), incubated on ice for 15 min and centrifuged as above. The pellet was resuspended in 10 ml 5 mM MgSO_4_, 1 mM EDTA and incubated for 15 minutes on ice before a final centrifugation step as above. Both supernatants were pooled, dialyzed against PBS and stored at 4°C.

Recombinant proteins were also expressed in the periplasm under osmotic stress in the presence of compatible solutes as described by Barth et al. [20]. Briefly, bacteria were grown overnight at 26°C in Terrific Broth (TB) (12 g l^-1 ^bacto-tryptone, 24 g l^-1 ^bacto-yeast-extract, 4 ml l^-1 ^glycerol) containing 100 μg ml^-1 ^ampicillin and 0.5 mM ZnCl_2_. The culture was diluted 30-fold in 200 ml of the same medium. When the OD_600 nm _of the culture reached 2.0, it was supplemented with 0.5 M sorbitol, 4% NaCl, 40 mM glycine betaine and incubated at 26°C for an additional 30–60 min. Expression was induced with 1 mM final concentration IPTG and growth for 6 h at 26°C. Cells were harvested by centrifugation at 30,000 × g for 10 min. The recombinant antibody fragments were isolated from the periplasmic space as described above. The periplasmic and osmotic shock fractions were pooled and dialyzed against PBS. Phenylmethylsulfonylfluoride (PMSF) was added to a final concentration of 1 mM.

### Purification of recombinant proteins

The human scFv and diabody antibody fragments were purified by IMAC using the His_6 _tag as described by Griffiths et al. [21]. Briefly, 10 ml columns (BioRad Polyprep chromatography columns) were packed with 500 μl Ni-NTA resin (Qiagen) and washed with five column volumes of PBS prior to loading with the recombinant proteins. The columns were washed with 10 column volumes of PBS containing 10 mM imidazol. Bound proteins were eluted with 250 mM imidazol and collected in 1-ml fractions. Protein concentrations were determined by spectrophotometry assuming that A_280 nm _= 1 corresponds to a scFv or diabody concentration of 0.7 mg ml^-1^.

Gel filtration was used for further purification. A Sephadex 200 column (Pharmacia) was equilibrated with PBS. ScFv or diabody antibody fragments were loaded and run at 1 ml min^-1^. Aprotinin (6500 Da), cytochrome C (12,400 Da), carbonic anhydrase (29,000 Da), bovine serum albumin (BSA) (66,000 Da) and Dextran Blue (2,000,000 Da) were used as molecular weight standards (Fluka).

### Enzyme linked immunosorbent assay (ELISA)

*S. mutans*, SAI/II antigen or BSA were coated on ELISA plates (Nunc) at a concentration of 1–10 μg per well in PBS overnight at 4°C. The plates were washed three times with PBS and blocked with 2% milk powder in PBS for 2 h at room temperature. The scFvs were tested either at a concentration of 1 μg per well or 100 μl per well of overnight-induced culture. Recombinant antibodies containing the MYC tag were detected with the murine 9E10 monoclonal antibody (ATCC CRL1729). Antibodies containing a His_6 _tag were detected using the murine anti-Penta-HIS antibody (Qiagen). The murine antibodies were detected with a goat-anti-mouse (Fc-specific) peroxidase-labeled antibody. The assays were developed with 3,3',5,5'-tetramethylbenzidine (TMB) (Sigma). Reactions were stopped by the addition of H_2_SO_4 _after 20 min and readings taken at OD_450 nm_. Between every incubation step, the plates were washed three times with PBS containing 0.05% Tween 20 and three times with PBS.

**Inhibition ELISA **was carried out by binding of chimeric scFv to SAI/II and replacement with an excess of mAb Guy13. Bound scFv were detected as described above using the murine 9E10 monoclonal antibody detecting the MYC-tag.

### Agglutination of *S. mutans*

Cultured *S. mutans *was divided into 20-μl aliquots and incubated with serial dilutions of bacterially expressed recombinant antibodies dia mGuy13 and diaD12, respectively for 2 d at 4°C or 1 h at 37°C on Lab-Tek II chamber slides (Nalge Nunc International). As negative control an unrelated human diabody was used. Excess medium was discarded and the cells were air-dried. The bacteria were counterstained with Gram solution (Diagnostica Merck). The slides were mounted with Immunofluor medium (ICN Biomedicals Inc) and photographed with a Zeiss Axioskob microscope.

## Authors contributions

MBK generated the diabody formats and performed all *in vitro *assays. MH did the upscaled expression of the antibodies. HS assisted in the analysis of data. JKCM assisted in the analysis and interpretation of the data especially in respect to the data obtained with the murine mAb Guy13. SB assisted in the interpretation of the data. RF assisted in the conception and revision of the project. RF* generated the human phage display libraries, developed the concept and supervised the project

## Figures and Tables

**Table 1:** Deduced amino acid sequence of the heavy and light chain variable regions of human scFv and diabody.

**scFv**	**FR1**	**CDR1**	**FR2**	**CDR2**
MuV_H_Guy13	QVKLQESGPDLVKPGASVKISCKASGYTFT	DYNIH	WVKQSRGKSLEWIG	YIYPYNGNTYYNQKFKN
MuV_L_Guy13	DIELTQSPAIMSASPGEKVTITC	SASSSVSYMH	WFQQKPGTSPKLWLY	STSNLAS
				
HuV_H_B10	QVQLQESGAEVKKPGLGEGLLQASGGTFS	RYALS	WVRQAPGQGLEWMG	GIIPIFGTTNYAQKFQG
HuV_L_B10	DIQMTQSPSSLSASVGDRVTITC	RASQGISNYLA	WFQQKPGKAPKSLIY	AASSLQS
				
HuV_H_D12	QVQLQEXGAEVKKPGESLKIXCKGSGYSFT	SYWIG	WVRQMPGKGLEWMG	IIYPGDXDTRYSPSFQG
HuV_L_D12	DIQMTQSPSTLSASIGDRVTITC	RASEGIYHWLA	WYQQKPGKAPKLLIY	EASRLQS
				
HuV_H_H6	QVQLQESGGGVVQPGRSLRLSCAASGFTFS	SYAMH	WVRQAPGKGLEWVS	YISSSGSYIYYADSVKG
HuV_L_H6	DIVMTQSPSSLSASVGDRVTITC	RASQGISNYLA	WFQQKPGKAPKSLIY	AASSLQS
**scFv**	**FR3**	**CDR3**	**FR4**	

MuV_H_Guy13	KATLTVDNSSTSAYMELRSLTSEDSAVYYCAT	YFDY	WGQGTTVTVS	
MuV_L_Guy13	GVPARFSGSGSGTSYSLTISRMEAEDAATYYC	HQRTSYPYT	FGGTKLEIKR	
				
HuV_H_B10	RVTIAADESTSTAYLELSSLRSEDTALYYCAK	SYDYVWGSYRPNEYGLDI	WGQGTMVTVS	
HuV_L_B10	GVPSKFSGSGSGTEFTLTISSLQPEDFATYYC	QELISYPLT	FGGGTKLEIKRA	
				
HuV_H_D12	QVTISADKSISTAYLQWSSLKASDTAMYYCAR	LGLQDDYVWGSXNWFDP	WGQGTLVTVSTS	
HuV_L_D12	GVPSRFSGSGSGTEFTLTISSLQPDDFATYYC	LQDFTYPRT	FGQGTKVEIKRA	
				
HuV_H_H6	RFTISRDNAKNSLYLQMNRLRAEDTAVYYCAR	DMAGTSYYYYYMDV	WGKGTLVTVSTS	
HuV_L_H6	GVPSKFSGSXSGTEFTLTISSLQPEDFATYYC	QELISYPLT	FGGGTKLEIKRA	

FR = antibody framework region, CDR = complementarity determining region

**Table 2:** Construction of human diabodies. Primer combinations and primer sequences

Template	V_L _amplification		V_H _amplification	
pHenIX human scFv B10	Vκ1 ApaL1	Jκ1 Not1	VH4 Sfi1/Nco1	JH3 for Sal1
pHenIX human scFv H6	Vκ1 ApaL1	Jκ1 Not1	VH6 Sfi1/Nco1	JH2 for Sal1
pHenIX human scFv D12	Vκ1 ApaL1	Jκ1 Not1	VH4 Sfi1/Nco1	JH2 for Sal1
pHenIX scFv mGuy13	mGuy13 ApaL1	mGuy13 Not1	VH4 Sfi1/Nco1	mGuy13 Sal1

Vκ1 ApaLI: 5'-TGAGCACACAGTGCACTCGACATCCAGATGACCCAGTCTCC-3'

Jκ1 Not1: 5'-GAGTCATTCTCGACTTGCGGCCGCACGTTTGATC/TTCCAC/GCTTGGTCCC-3'

V_H_4 Sfi1/Nco1: 5'-GTCCTCGCAACTGCGGCCCAGCCGGCCATGGCCCAGGTGCAGCTGCA/ GGAGTCGGG-3'

V_H_6 Sfi1/Nco1: 5'-GTCCTCGCAACTGCGGCCCAGCCGGCCATGGCCCAGGTACAGCTGCA/ GCAGTCAGG-3'

J_H_3 Sal1: 5'-GAGTCATTCTCGTGTCGACACGGTGACCATTGTCCC-3'

J_H_2 Sal1: 5'-GAGTCATTCTCGTGTCGACACAGTGACCAGGGTGCC-3'

mGuy13 ApaL1I:5'-TGAGCACACAGTGCACTCGACATCGAGCTCACTCAGTCTCC-3'

mGuy13 Not1: 5'-TTTTCCTTTTGCGGCCGCCCGTTTTATTTCCAACTTTGT-3'

mGuy13 Sal1: 5'-GAGTCATTCTCGTGTCGACACGGTGACCGTGGTGCCTTGGCCCCAGTAGTCAAAGTAGGT-3'
